# A cluster randomized trial to assess the impact of opinion leader endorsed evidence summaries on improving quality of prescribing for patients with chronic cardiovascular disease: rationale and design [ISRCTN26365328]

**DOI:** 10.1186/1471-2261-5-17

**Published:** 2005-06-27

**Authors:** Sumit R Majumdar, Finlay A McAlister, Ross T Tsuyuki

**Affiliations:** 1 Department of Medicine, University of Alberta, Edmonton AB and the Institute of Health Economics, Edmonton AB, Canada

## Abstract

**Background:**

Although much has been written about the influence of local opinion leaders on clinical practice, there have been few controlled studies of their effect, and almost none have attempted to change prescribing in the community for chronic conditions such as heart failure (HF) or ischemic heart disease (IHD). These two conditions are common and there is very good evidence about how to best prevent morbidity and mortality – and good evidence that quality of care is, in general, suboptimal. Practice audits have demonstrated that about one-half of eligible HF patients are prescribed ACE inhibitors (with fewer still reaching appropriate target doses) and less than one-third of patients with established IHD are prescribed statins (with many fewer reaching recommended cholesterol targets). It is apparent that interventions to improve quality of prescribing are urgently needed. We hypothesized that an intervention that consisted of patient-specific one-page evidence summaries, generated and then endorsed by local opinion leaders, would be able to change prescribing practices of community-based primary care physicians.

**Methods (study design):**

A pragmatic single-centre cluster randomized controlled trial comparing an opinion leader-based intervention to usual care for patients with HF or IHD. Randomization will be clustered at the level of the primary care physician; as the design effect is anticipated to be negligible, the unit of analysis will be the patient. Patients with HF or IHD (not receiving ACE inhibitors or statins, respectively) will be recruited from community pharmacies and allocated to intervention or usual care based on the randomization status of their primary care physician. The primary outcome is improvement in prescription of proven efficacious therapies for HF (ACE inhibitors) or IHD (statins) within 6 months of the intervention.

**Conclusion:**

If the methods used in this intervention are found to improve prescribing practices, similar interventions could be designed for other chronic conditions dealt with in the outpatient setting.

## Background

### Opinion leaders – an untapped resource for quality improvement

Local opinion leaders are able to influence the practice of other physicians because they are well-known, respected, and trusted to evaluate medical innovations within the local context [1-4]. Because they influence patterns of practice in the community, and may accelerate the uptake of knowledge, their participation in any program of quality improvement is essential. Certainly, surveys of physicians [5,6] have consistently confirmed the importance of colleagues and local consultants on individual patterns of practice. Yet, the use of local opinion leaders to influence physician practice has only been tested in six randomized controlled trials [1,2]. While two of these trials [3,4] demonstrated an important impact upon practice, both assessed labour-intensive, expensive, hospital-based educational interventions spearheaded by a small number of opinion leaders (four in one study, 16 in the other) for conditions treated in a hospital setting (delivery by cesarean section, treatment of acute myocardial infarction). Although the use of opinion leaders to influence the outpatient management of common conditions holds great promise, this is yet a hypothesis to be rigorously tested [7].

### Underuse of proven medications for cardiac diseases is common

In fact, most patients with chronic conditions (such as heart failure [HF] or atherosclerotic ischemic heart disease [IHD]) are treated as outpatients. Previous practice audits have documented significant "care gaps" between the available evidence and actual practice (e.g., only half of eligible patients with HF receive an ACE inhibitor [7,8], despite overwhelming evidence of benefit [7,8], and those who are prescribed an ACE inhibitor are usually given doses below those tested in clinical trials [10]; less than a third of patients with established IHD receive lipid-lowering therapy [7,10,11], despite multiple trials [7,12,14] showing benefit, and of those prescribed lipid-lowering therapies, only 15% achieve recommended cholesterol goals [11]). As these two conditions represent an important burden of illness for the community [15], closure of these care gaps should be a public health priority. Moreover, we believe methods need to be developed that can reliably and efficiently improve the quality of care for these high-risk populations [7].

### Community-based interventions must be simple and practical

When testing the effect of opinion leader influence in the outpatient setting, we must keep in mind the potential generalizability and applicability of any proposed intervention. Thus, the focus must be on a practical means of incorporating opinion leaders into practice, because we know that opinion leaders may exert considerable influence on community practice. In addition, previous studies have established that the methods of information transfer most favoured by physicians are one-page summaries of guidelines or evidence [5,17]. Moreover, other work has suggested that when the transfer of clinically relevant information is directly linked to a specific patient encounter with a specific recommendation (e.g., real-time reminders, whether manual or computerized) there is a much greater likelihood of affecting change [2,7,18]. The ideal way to create such a scenario would be to have a "real-time" reminder generated in the physicians' office and linked to the actual clinical encounter. This is not currently practical or feasible within the vast majority of community practices in Canada – most of these practices are still paper-chart based, and even of those practices that have electronic medical records and some capacity for computerized decision support, most are not linked to other databases such as vital statistics, hospital discharge databases, or pharmacy dispensing records.

### Potential role of community pharmacies and pharmacists

Prior research, and our own ongoing work in other clinical arenas, leads us to believe that the community pharmacy may be a reasonable location to generate the requisite linkages needed to undertake an intervention to improve quality of outpatient prescribing as well as a convenient site for unobtrusively recruiting patients [19]. This is because patients with chronic conditions can be identified by "marker medications" at the time of presentation to their pharmacy (more than 90% of HF patients are prescribed loop diuretics [8], and almost 100% of patients with known IHD are given prescriptions for short-acting nitrates [20]), and because pharmacists already interact on a regular basis with both prescribing physicians and their patients. Therefore, the community pharmacy may serve as a potential site for patient recruitment and education or intervention. For example, the SCRIP study used 54 community pharmacies in Western Canada, and demonstrated improvements in lipid management in a 675 patient randomized trial [19]. This pharmacy network has evolved and expanded to bring together community pharmacists, physicians and other healthcare professionals into broad-based community-level studies through The Epidemiology Coordinating and Research (EPICORE) Centre and its subsidiary, the Centre for Community Pharmacy Research and Interdisciplinary Strategies (COMPRIS) at The University of Alberta.

### Evidence-based interventions to improve quality of prescribing

With these basic considerations in mind, we designed an evidence-based multi-faceted intervention to improve the quality of prescribing in the community. We propose having locally-nominated opinion leaders generate and endorse one-page evidence summaries for two common and chronic cardiovascular conditions. These evidence summaries, linked with specific patient-level medication profiles (generated at the community pharmacy), will be distributed to practicing physicians and attached to their patients' chart. Our hypothesis is that this will act as both a source of credible and convincing information and a specific reminder for action at the next patient encounter. Our study is designed to test this hypothesis, by assessing the impact of this intervention on the quality of prescribing for patients with heart failure or ischemic heart disease.

## Methods

### Purpose

The purpose of this study is to determine whether "evidence summaries," generated and endorsed by local opinion leaders, can improve the prescribing of proven efficacious therapies by primary care physicians for their patients with chronic cardiovascular diseases.

### Objectives

This study has three main objectives:

1. To determine whether evidence summaries generated and endorsed by local opinion leaders can increase the prescription of efficacious therapies for patients with HF or IHD.

2. To compare the effectiveness of the proposed intervention with two different conditions, to begin to understand the potential for generalizability across different diseases and conditions.

3. To evaluate whether distribution of local opinion leader endorsed evidence summaries, through community pharmacies, is a viable quality improvement tool within the constraints of current healthcare delivery systems.

### Study design

A single centre pragmatic cluster randomized controlled trial in which primary care physicians and their patients will be randomly allocated to **intervention **(relevant single page evidence summary endorsed by local opinion leaders and a copy of the patient's current medication profile faxed back to the primary care physician) or **control **(only the patient's current medication profile faxed back to the primary care physician). To prevent the potential for "contamination" within an individual physicians' practice (e.g., allocation of an intervention and a control patient to the same physician) a modified form of cluster allocation will be undertaken. Specifically, if a physician is randomized to the intervention arm for IHD, her other IHD patients that present to a participating pharmacy will also be subject to the IHD intervention, to a maximum of five patients. In addition, she will also act as her own control, because she will be assigned to the control arm for her HF patients, again to a maximum of five patients. For a schematic of the overall study design, please see Additional File: 1.

### Identifying opinion leaders and generating evidence summaries

All primary care physicians within Capital Health (the greater Edmonton metropolitan area, Alberta, Canada) were mailed a one-page survey (based on a previously validated opinion leader nomination instrument [3,4,21]) asking them to nominate local colleagues who best match standard descriptions of "educationally influential" opinion leaders (see Additional File: 2). The overall response rate was 30% (225 of 788 surveys returned), typical of the response rates for physician surveys. Five opinion leaders were overwhelmingly identified by their peers (1 physician for HF only, 1 physician for IHD only, and 3 physicians nominated for both HF and IHD; 3 opinion leaders were cardiologists and 2 were general internists) and were approached to participate in the development of the one-page evidence summaries for HF and for IHD. All 5 nominated opinion leaders participated in the development of the intervention materials. The survey instrument also informed primary care physicians about the study (in general terms) and provided them the option to "opt-out" of having themselves or their patients recruited or enrolled in the study – only 19 physician respondents declined to participate. Physician survey non-respondents (i.e., those who did not explicitly and actively opt-out of the study) will be considered eligible for the study, although any physician will be able to withdraw themselves and their patients at any time.

The data for the evidence summaries will be initially generated by experts in evidence-based medicine and cardiovascular care using standardized techniques for research synthesis [22]; the opinion leaders and study investigators will then ensure that treatment recommendations are consistent with available clinical practice guidelines, and then carefully incorporate this material into the one-page evidence summaries. The final content and format of these summaries will be arrived at by standard consensus methods, and these evidence summaries will form the core of our intervention.

### Description of the intervention

The intervention consists of a disease-specific and patient-specific one-page evidence summary. It will be a patient-specific letter addressed to the patients' primary care physician, along with a description of the potential risks of undertreatment and current evidence-based treatment recommendations. The letter will be signed and endorsed by all 5 of the study opinion leaders. Examples of the IHD and HF letters are provided in Additional Files: 3 and 4.

Accompanying the letter will be the most recent pharmacy record of medications dispensed to the study patient. It is intended that the evidence summary and the pharmacy medication profile will become part of the patients' medical record and act as a reminder or prompt at the *next *patient visit. These materials will be faxed to the primary care physician from the patients' community pharmacy.

### Controls ("usual care")

Physicians of control patients will only receive the pharmacy medication dispensing record. This will be done for two reasons. First, to disentangle the effects of having a complete medication record (in and of itself, a departure from "usual care") versus the impact of the evidence summaries. Second, to control for the possibility of any attention-related or study-related "Hawthorne effects" – all patients and all physicians will receive the same number of contacts and follow-ups, and all that will differ between the experimental arms is the content of the intervention. Again, control materials will be faxed from the patients' community pharmacy. It should be noted here that "control," as we have defined it, is actually more rigorous than the current standard of care; it might also be argued simply providing a medication profile would be sufficient in an of itself to change practice, although a recent systematic review concluded that this would not be the case [2].

### Study setting

Capital Health (Edmonton, Alberta) is one of the largest integrated health service delivery organizations in Canada. It provides comprehensive health services for almost one million people and has an annual budget of almost two billion dollars Canadian. Primary care is delivered by approximately 800 fee-for-service physicians. Community dwelling patients with cardiovascular disease (either HF or IHD) will be recruited from a convenience sample of 40 different community pharmacies, some of which have previously participated in pharmacist-based research studies. It should be noted that, at the time of study design, none of these pharmacies were electronically linked to either physicians' practice records or any form of patients' medical charts.

### Subjects

Involvement of primary care physicians has already been described, and we potentially will be able to draw subjects from the population-base of all fee-for-service physicians in the entire region. Patients (and their prescribing physicians) will be identified by community pharmacists from lists obtained from their computerized dispensing records. Patients with a prescription for a "marker" medication (a loop diuretic for the proxy diagnosis of HF; a short acting nitrate for the proxy diagnosis of IHD) who are not currently taking the respective study medications of interest will be notified by their own pharmacist about the study and informed that (with their permission) a member of the research team will contact them with more information about the study. A research assistant (pharmacist) will then initiate telephone contact once potential eligibility has been determined, and obtain verbal consent from eligible patients to access their pharmacy records twice (study entry and 6-month followup) for the purposes of the study. It should be noted that these patient's pharmacists, providing routine services in the community, already have access to their pharmacy records. Specific criteria for inclusion and exclusion include the following:

#### Inclusion criteria

Patients with HF or IHD who are not currently taking the study medications of interest (ACE inhibitors/angiotensin receptor blockers for HF or statins for IHD), and whose primary care physician of record is part of the study. For patients who happen to be eligible for both HF and for IHD, only one condition will be selected at random.

#### Exclusion criteria

Patients who decline enrollment, or who are unable or unwilling to give informed consent, or who have previously taken the study medications according to dispensing records, or who have a documented allergy or intolerance to study medications according to pharmacist records, or who are in long-term care facilities or institutions, or who do not confirm on the basis of self-report that they have a diagnosis of either HF or IHD, or whose primary care physician has already contributed 5 patients to the study.

### Allocation to experimental arms

Simple randomization will occur at the level of the primary care physician, before patient recruitment and enrollment begins, using a computer-generated sequence with allocation concealment. Each participating physician will be randomly allocated to intervention or control arm for HF; physicians allocated to intervention for HF will be automatically assigned to the control arm for IHD, and vice versa. This design prevents "contamination" within an individual physicians' practice (e.g., having one intervention HF patient and one control HF patient), while simultaneously increasing study efficiency (i.e., physicians can contribute patients to both the HF and IHD arms of the study, thus potentially reducing the overall number of physicians required). Furthermore, no one physician will be permitted to contribute more than 5 patients to the study, to minimize any issues related to the potential for physician-level clustering of study outcomes [23,24].

That said, this might be considered a form of "cluster" randomization [2,23,24]. However, on a population-wide basis we anticipate that most regional physicians will contribute no patients, and the majority of physicians will contribute no more than 1 study patient. This has been the case in previous studies we have recently undertaken using similar recruitment and allocation methods [19,25]. Furthermore, by our study design, no physician will be able to contribute more than 5 patients to the study as a whole. Therefore, the "design effect" should be negligible, particularly since previous studies have demonstrated that important cluster effects do not come into play until the physician-to-patient ratio exceeds 1-to-5, and we expect our ratio to be close to 1-to-1; thus, all sample size and analytic considerations will be based on the patient as the unit of analysis and the unit of causal inference [23-25].

### Outcome measures

The primary outcome measure will be the "improvement" of prescribing for efficacious therapies in patients with a chronic cardiovascular disease within 6 months of the intervention. By study design, none of the study patients will be taking the medications of interest. For HF, starting any ACE inhibitor or angiotensin receptor blocker will be considered a positive outcome. For IHD, starting any statin will be considered a positive outcome. For the primary outcome all positive study-related medication changes will be pooled for an overall estimate of effect, compared with usual care controls.

The main secondary outcomes will be condition-specific "improvement" in prescribing after 6 months. In addition, we will attempt to assess optimization of dosage for each of the medications prescribed (i.e., ACE inhibitors or angiotensin receptor blockers and statins). We will also evaluate patient adherence (using prescription refill rates based on dispensing records) and examine the potential influence of age and sex on outcomes.

### Study procedures and data collection

The primary source of data for the study will be the patient-level medication profiles generated at each community pharmacy. All data will be collected by a research assistant traveling to each pharmacy. Data will be collected using a standardized abstraction instrument. Only data without unique personal identifiers, but with a unique study ID# (and therefore anonymized), will be entered into a secure database housed at the EPICORE Centre, University of Alberta. Investigators will be blinded to physician- and patient-level unique identifiers and investigators and study patients will be masked to allocation status. Primary care physicians themselves cannot be blinded to allocation status. Follow-up data collection is scheduled at 6-months, again based on patient-level medication profiles generated by participating pharmacists, and follow-up data will be collected without knowledge of allocation status in an independent and blinded fashion. All statistical analyses will be conducted by an independent statistician also masked to allocation status.

Covariates that will be collected include age, sex, and self-reported medical diagnoses. Using medication profiles, we will also be able to generate a previously validated measure of comorbidity, the Chronic Disease Score. This measure has been previously demonstrated to provide valid and reliable risk adjustment for comorbidity or case-mix, and it is able to predict long-term morbidity, hospitalization, mortality, and health care utilization [26].

The time period chosen for outcome assessment (6-months) was selected as the vast majority of HF patients (over 80% [10]) are seen at least every 6 months and a poll of general internal medicine specialists and cardiologists at the University of Alberta confirmed that most IHD patients are also seen within this same time period. Data collation, quality assurance, and statistical analyses will be carried out at the EPICORE Centre.

### Statistical analyses

Although the physician will be the unit of allocation, the patient is the unit of analysis and causal inference. This is justified by the anticipated small design effect, and the fact we expect that the outcomes for individual patients to be clinically and statistically independent of each other – because each intervention is itself both patient- and condition-specific. The main analysis will be a comparison of the proportion of all patients who successfully achieve the primary outcome ("improved" prescribing of efficacious therapies, as described above) at 6-months. This will be tested using the chi square statistic. There will be no planned interim analyses, and we will consider a p-value <0.05 to be statistically significant. Secondary analyses will compare the proportions of patients who successfully achieve the primary outcome in each of the two cardiovascular conditions (HF and IHD), to determine whether observed effects are consistent across different disease states. Two prespecified subgroups (male vs. female and age less than 70 years vs. 70 years of age and older) will also be considered. Secondary outcomes will be studied in an analogous fashion, and will be considered only exploratory in nature, and as such, comparisons will be made without corrections for multiple testing.

In order to investigate what factors are associated with changes in the primary outcome (our dependent binary variable), and to control for the possibility of potential imbalances in patient-level characteristics induced by our randomization scheme, multivariable logistic regression analyses will be used to examine those variables that are deemed to be clinically important (i.e., age, sex, diabetes status) or that differ statistically at a p-value <0.10 between experimental arms. In addition, to examine the remote possibility of "cluster-associated" study design effects, two secondary sensitivity analyses will be considered: first, the main analysis will be repeated using the physician as the unit of analysis [2,23,24]; and second, the aforementioned logistic regression models will be re-analyzed using generalized estimating equations to control for the potential lack of statistical independence among patients treated by the same study physician [2,23,24].

### Sample size considerations

Using surveys of physicians and policy-makers, we determined that the "minimal" clinically important difference for this particular intervention to be considered both useful (and perhaps) fundable on an ongoing basis, was a 20% improvement over and above usual care. By design, use of any of the study medications at baseline will be zero. After 6 months, we assume no more than 10% of control patients will have started a study medication. Acknowledging that a 20% absolute increase in the primary outcome (starting a study medication, either an ACE inhibitor or angiotensin receptor blocker [HF] or a statin [IHD]) would be a clinically important effect size, setting the α error rate at 0.05 (2-sided), and the β error at 0.20 (power 80%), a total sample size of 140 will be required. Allowing for losses to follow-up, the ability to examine each of the conditions separately, and the possibility of a very small design effect associated with patient clustering, the total sample size has been adjusted upwards to 160 patients.

### Ethical considerations

The protocol and procedures as described have received institutional approval from the Health Research Ethics Board of the University of Alberta. In addition, any individual physician or patient will have the opportunity to withdraw from the study at any time, and none of the investigators or analysts will have access to any physician-identifiable or patient-identifiable data. The funding for the study is from two peer-reviewed grants. The funding sources (Alberta Heritage Foundation for Medical Research and the Institute of Health Economics, Edmonton, Alberta) had no role in the design, conduct, analysis, interpretation, or reporting of the study and will not have access to the data. Finally, it should be noted that the 5 opinion leaders received no compensation (financial or otherwise) whatsoever for their participation in the study or endorsement of the evidence summaries.

## Discussion

Herein, we have reported the background, rationale, and study protocol for a pragmatic cluster randomized controlled trial of an intervention that will test the hypothesis that opinion leader-generated and endorsed one page evidence summaries that are patient-specific will be able to improve the quality of cardiovascular medication prescribing. The opinion leader literature is sparse at best, and yet the potential role and educational influence of these important physician champions is vastly underappreciated. To our knowledge, this will be the only rigorous study of the "opinion leader hypothesis" undertaken outside the acute care or hospital setting. In addition, we will be able to gather valuable data about the potential role for community pharmacies and pharmacists in improving the quality of care for patients with chronic diseases that (by definition) require careful and longterm polypharmacy.

Perhaps a more ideal study could be undertaken in a more hospitable setting, such as a large managed care organization or a large academic medical centre – one that has a captive population and where all health providers have access to a universal electronic medical record that is directly linked to pharmacy dispensing databases and where there is potential for computerized decision support with the capacity to generate real time reminders and prompts. Indeed, this has been tested in a randomized trial for outpatients with chronic HF or chronic IHD (an almost identical population in most respects to the one we are enrolling), and it has been found wanting [27]. It could be that information technology alone will be unable to improve the quality of care if the treatment recommendations are not first endorsed by local opinion leaders. This possibility, as well as the fact that most community-based practices are years to decades away from having clinically useful and integrated electronic health information systems, will mean that the results of our study will likely have broader implications than for just the two conditions we chose to study.

## Conclusion

If we are able to demonstrate that opinion leaders can change clinical practice in the community, then larger multi-centre studies of different conditions (e.g., controller medications in asthma, antiresorptive therapies in osteoporotic patients with fractures, antihypertensive agents in people with diabetes) will need to be undertaken.

## Abbreviations

IHD, ischemic heart disease; HF, heart failure; ACE inhibitor, angiotensin-converting enzyme inhibitor

## Competing interests

The author(s) declare that they have no competing interests.

## Authors' contributions

All of the authors contributed equally to all aspects of the manuscript and fulfill all standard criteria for authorship. Sumit Majumdar also drafted the first version of the manuscript and will act as guarantor.

## Pre-publication history

The pre-publication history for this paper can be accessed here:



## Supplementary Material

Additional File 1 Overview of Study Design Click here for file

Additional File 2 Opinion Leader Survey Instrument Click here for file

Additional File 3 Example of the Evidence Summary for Heart Failure Click here for file

Additional File 4 Example of the Evidence Summary for IHD Click here for file
