# Supplementary material for: A cluster randomized trial to assess the impact of opinion leader endorsed evidence summaries on improving quality of prescribing for patients with chronic cardiovascular disease: rationale and design [ISRCTN26365328]
Source: BMC Cardiovasc Disord. 2005 Jun 27;5:17. doi: 10.1186/1471-2261-5-17 (PMC1175844; doi:10.1186/1471-2261-5-17)
Supplement: Additional File 1 — Overview of Study Design [file 1471-2261-5-17-S1.doc]

**Figure 1. Overview of Study Design**

# Primary Care Physicians Surveyed

# and Opinion Leaders Nominated

**Randomization**

## CHF CHF IHD IHD

### Intervention control control intervention

# Entered into Study According to Disease State

# and Allocation Status of their Primary Care Physician

# Eligible, Agree to Inclusion in the Study, and Provide Consent

# Potentially Eligible Subjects Contacted by Research Team

**for Inclusion in the Study**

# Potentially Eligible Subjects Notified of Study by Pharmacist

# Screening of Medication Database by Pharmacist,

# Using Marker Medications (loop diuretics or nitrates)
