# Supplementary material for: A cluster randomized trial to assess the impact of opinion leader endorsed evidence summaries on improving quality of prescribing for patients with chronic cardiovascular disease: rationale and design [ISRCTN26365328]
Source: BMC Cardiovasc Disord. 2005 Jun 27;5:17. doi: 10.1186/1471-2261-5-17 (PMC1175844; doi:10.1186/1471-2261-5-17)
Supplement: Additional File 2 — Opinion Leader Survey Instrument [file 1471-2261-5-17-S2.doc]

**Figure 2. Opinion Leader Survey Instrument**

Dear Dr. ________________,

We are trying to study novel methods for providing clinically useful and relevant continuing medical education (CME) to busy physicians. We believe that high quality CME consists of practical information that can be directly applied to one’s patients and it should come from local experts that are well-known and trusted.

Pharmacies throughout Capital Health are generating patient lists for two conditions - **ischemic heart disease** and **congestive heart** **failure**. These patients will serve as real-world “examples” for a new case-based CME program. Our goal is to generate evidence-based messages with these patients in mind. To make these messages relevant, we need to know whom the physicians are that **you** look to for guidance and advice. Completing this one-page survey is all that will be asked of you for this study.

**We want to know whom the physician(s) are that:**

- you usually approach about difficult cases
- you consider good teachers (e.g., up-to-date, express themselves clearly)
- you know will take the time to help you out, no matter how busy they are
- always treat you as a respected colleague
- you believe are expert clinicians that are competent and caring
- you would send a family member to

Please name the physicians who best fit the above description, with respect to:

**Ischemic heart disease**	**#1_____________________________________**

**#2_____________________________________**

**Congestive heart failure	#1_____________________________________**

**#2_____________________________________**

Please return this survey by fax to **780-407-XXXX**. Thank you very much for your time and consideration. All information that is collected will be kept strictly confidential. If you have any questions or concerns please fax us or contact us directly at **780-407-XXXX**.

If you do not want to participate in this region-wide CME program, please sign here **________________________**, and return this survey to our office by fax.

Yours sincerely,
