# Supplementary material for: A cluster randomized trial to assess the impact of opinion leader endorsed evidence summaries on improving quality of prescribing for patients with chronic cardiovascular disease: rationale and design [ISRCTN26365328]
Source: BMC Cardiovasc Disord. 2005 Jun 27;5:17. doi: 10.1186/1471-2261-5-17 (PMC1175844; doi:10.1186/1471-2261-5-17)
Supplement: Additional File 3 — Example of the Evidence Summary for Heart Failure [file 1471-2261-5-17-S3.doc]

**Figure 3. Example of the Evidence Summary for Heart Failure**

January 1^st^, 200X

Dr. PC Provider

123-456 Avenue

Edmonton, AB, Canada

Dear Dr. Provider,

Based on some clinical data and a medication profile, your patient _________________ likely has a history of **congestive heart failure**. As you know, most patients like this will derive significant benefit by using an ACE inhibitor. You may also be aware that there is new evidence suggesting that:

- ACE inhibitors are beneficial for all patients – with any systolic dysfunction or **any** **degree** of heart failure, from the mildest to the most severe.
- The higher the dose the better – for example, lisinopril 30mg qd is significantly better at preventing hospitalization or death than 2.5mg qd.
- ACE inhibitors are still the first line agents for preventing CHF morbidity and mortality, although angiotensin receptor blockers are beneficial for the few patients unable to tolerate an ACE inhibitor due to cough.

**Therefore, we have started recommending the following approach routinely:**

1. An evaluation to confirm and evaluate the presence of heart failure, including an echocardiogram or gated pool scan (MUGA) if one has not been performed.
2. **Starting an ACE inhibitor**, if your patient has ever had any symptoms of heart failure or the echocardiogram/MUGA shows any left ventricular dysfunction.
3. **Titrating up the ACE inhibitor dose**, towards the target doses used in recent trials (e.g., in alphabetical order, captopril 6.25mg to 50mg tid; enalapril 2.5mg to 10mg bid; lisinopril 2.5mg to 30mg qd; quinapril 2.5mg to 40mg qd; and ramipril 2.5mg to 10mg qd). Because of symptomatic hypotension or progressive renal impairment, not everyone may be able to tolerate these higher doses.

These recommendations reflect our current practice, and are based on recently published studies and guidelines. If you have any further questions or concerns, or would like any of the literature supporting these recommendations, please contact us…

Yours sincerely,

**Opinion Leader #1,	Opinion Leader #2,	Opinion Leader #3,**

**Opinion Leader #4,	Opinion Leader #5**
