# Supplementary material for: A cluster randomized trial to assess the impact of opinion leader endorsed evidence summaries on improving quality of prescribing for patients with chronic cardiovascular disease: rationale and design [ISRCTN26365328]
Source: BMC Cardiovasc Disord. 2005 Jun 27;5:17. doi: 10.1186/1471-2261-5-17 (PMC1175844; doi:10.1186/1471-2261-5-17)
Supplement: Additional File 4 — Example of the Evidence Summary for IHD [file 1471-2261-5-17-S4.doc]

**Figure 4. Example of the Evidence Summary for IHD**

January 1^st^, 200X

Dr. PC Provider

123-456 Avenue

Edmonton, AB, Canada

Dear Dr. Provider,

Based on some clinical data and a medication profile, your patient ______________________ likely has a history of **ischemic heart disease** and therefore has more than a 30% 10-year risk of a cardiovascular event. As you know, most patients like this derive significant benefit from lipid lowering treatment with a "statin". You may also be aware that there is new evidence suggesting that:

- Statins consistently reduce mortality and morbidity, including the need for revascularization, in patients with **any degree** of ischemic heart disease or **any degree** of dyslipidemia.
- Optimal benefit from statin therapy is achieved if the LDL cholesterol can be brought below 2.5 mmol/L.

**Therefore, we have started recommending the following approach routinely:**

1. An evaluation to detect the presence of any atherosclerosis (if not already established, and a fasting lipid profile if one has not been done in the last year).
2. **Starting a statin of your choice** in all patients with coronary artery disease, cerebrovascular disease, peripheral vascular disease, or diabetes mellitus.
3. **Follow-up tests in 6 weeks**, including a repeat LDL and liver function tests.
4. **Titrating up the statin dose**, to achieve an LDL level below 2.5 mmol/L.

These recommendations reflect our current practice, and are based on recently published studies and guidelines. If you have any further questions or concerns, or would like any of the literature supporting these recommendations, please contact us…

Yours sincerely,

**Opinion Leader #1,	Opinion Leader #2,	Opinion Leader #3,**

**Opinion Leader #4,	Opinion Leader #5**
